# Age-related comparisons by sex in the domains of aerobic physical activity for adults in Scotland

**DOI:** 10.1016/j.pmedr.2015.12.013

**Published:** 2015-12-30

**Authors:** Tessa Strain, Claire Fitzsimons, Charlie Foster, Nanette Mutrie, Nick Townsend, Paul Kelly

**Affiliations:** a The Physical Activity for Health Research Centre, Institute for Sport, Physical Education and Health Sciences, University of Edinburgh, Scotland, United Kingdom; b The British Heart Foundation Centre on Population Approaches for Non-communicable Disease Prevention, Nuffield Department of Population Health, University of Oxford, England, United Kingdom

**Keywords:** PA, physical activity, MVPA, moderate and vigorous physical activity, SHeS, Scottish Health Survey, MET, Metabolic Equivalent of Task, Physical activity, Exercise, Adult, Health surveys, Humans, Leisure activities, Sports, Walking

## Abstract

**Objective:**

To investigate the age-related differences in the contributions of the domains of physical activity (PA) for men and women in Scotland who met the current PA guidelines or who were insufficiently active.

**Methods:**

We analysed data from the 2013 Scottish Health Survey (4885 adults (≥ 16 years)). Average weekly minutes of moderate or vigorous PA (MVPA) and the relative contributions to total MVPA were calculated for the domains of: walking, cycling, domestic, leisure, occupational, outdoor, non-team sport, team sport, and exercise & fitness. We performed linear regression analyses to assess differences by 10-year age group, stratified by sex and activity status (1–149 or ≥ 150 min of MVPA per week). These were repeated excluding occupational activity due to concerns with its measurement.

**Results:**

For the 64.3% of the sample that met the guidelines, occupational activity was the most prevalent domain accounting for 18–26% of all MVPA for those under 65 years. When excluded, there was no age-related decline in total MVPA (p > 0.05). For the 18.6% of the sample that reported 1–149 min of MVPA per week, domestic activity was the most prevalent domain. Across both sexes and activity statuses, exercise & fitness declined with age and walking was most prevalent in the oldest age group.

**Conclusion:**

The domains in which adults in Scotland undertake MVPA vary by age group. Policies designed to increase PA should take this into account. Our findings challenge current thinking on age-related changes in activity, with the exclusion of occupational activity mitigating any age-related decline in MVPA.

## Highlights

• Domains of physical activity vary by age for men and women in Scotland.
• Domestic activity was the most prevalent domain amongst the insufficiently active.
• Walking and occupational activitywere most prevalent for the sufficiently active.
• Occupational activity may distort our understanding of the age-related variations.
• Data from the nationally representative Scottish Health Survey

## 1 Introduction

Increasing physical activity (PA) levels is a successful and sustained policy priority in Scotland (The Scottish Government, 2014b). Progress is primarily monitored by the proportion of the population meeting the aerobic component of the guidelines (150 min moderate activity, or 75 min of vigorous activity or equivalent combination per week) (Department of Health, 2011), as reported annually by the Scottish Health Survey (SHeS). In 2013, 64% of the adult population in Scotland met these guidelines, an increase of 2% on the previous year. The current UK PA guidelines for adults also include statements on muscle strengthening and sedentary time, but specific indicators and policies for these modes are yet to be developed. This paper focusses solely on aerobic PA.

The SHeS records PA under the domains of domestic, occupational, sport and exercise, and walking. This information is important from a public health perspective as it provides the context in which PA is undertaken, potentially informing better intervention and policy design.

In England, Bélanger et al. (2011) found considerable age-related differences in the relative contributions of the domains of PA amongst adults who met the previous guidelines (30 min of moderate of vigorous PA (MVPA) on 5 days of the week). For example, the contribution of sports was negligible amongst older adults. Walking accounted for 26–42% of total MVPA in men and 37–45% in women and was the largest contributor for all age groups in both sexes, apart from in men aged 35–54 for whom occupational is. This highlights the need for interventions to be specific to the demographic characteristics of the target group. Whether the situation is the same in Scotland and with respect to the current PA guidelines is unknown.

This paper addresses this knowledge gap by providing Scottish-specific data on the age-related differences in the domain-specific contributions to total MVPA for men and women in Scotland. In addition, we provide a more in depth analysis than that of Bélanger et al. (2011) in four ways. Firstly, we present the absolute contributions, in addition to the relative contributions, of the domains of PA. This provides a fuller picture of where interventions are best targeted. Secondly, we performed the analyses on those who do not meet the current PA guidelines thus helping to identify potential domains to target and increase the proportion of adults meeting the PA guidelines. Thirdly, we ran our analyses both with and without the domain of occupational activity due to concerns that the measurement of this domain may distort the overall picture. Lastly, we performed statistical tests to assess whether the differences identified are statistically significant. Based on the results of Bélanger et al. (2011), we expected to see variations by age in the contributions of the domains of PA for men and women who met the guidelines. We were uncertain as to whether this would be the case for those who did not meet the guidelines.

## 2 Methods

### 2.1 Data source

We acquired the 2013 SHeS individual level dataset from the UK Data Archive (ScotCen Social Research) on 5th Feb 2015. The SHeS is designed to be nationally representative of the population living in private households in Scotland. The main survey consists of a computer aided personal interview during which PA data are collected. These are carried out over the whole year. Further information on the SHeS can be found in Corbett et al. (2014).

### 2.2 Questionnaire

The SHeS asks about PA in four domains in the 28 days prior to interview: (1) home-based activities (housework, gardening, building work and do-it-yourself home maintenance); (2) activity at work; (3) sports and exercise; and (4) walking. Further information can be found in the 2013 SHeS main and technical reports (Corbett et al., 2014, Hinchliffe, 2014). There have been no assessments of the questionnaire's validity or reliability to date but it is used to as the main source of data to inform Scottish PA policy. The similar but not identical Health Survey for England questionnaire demonstrated moderate convergent validity in comparison to accelerometry (Scholes et al., 2014). Average weekly time spent in these domains was converted into sub-domains developed from Bélanger et al. (2011). Activities reported under sports and exercise were allocated to leisure pursuits, outdoor pursuits, cycling, non-team sport, team sport or exercise & fitness but with cycling as independent sub-domain (see Supplementary material).

We used the same method for assigning intensity to the reported activities as used to derive the population estimates for the proportion meeting the guidelines in the SHeS annual reports. Only activities that are of at least moderate intensity count towards the PA guidelines and therefore were included in these analyses. Briefly, this excluded light housework, slow or steady average paced walks, and certain sport and exercise activities considered of light intensity such as snooker or darts were excluded. Heavy housework, brisk or fast paced walks and any occupational activity were considered of moderate intensity. Other sport and exercise activities were either categorised as moderate or vigorous in all situations, or were dependent on the answer to a follow up question that asked whether the activity makes the participant breathe faster, feel warmer or sweat to distinguish between the two intensity levels. These were based on the standardised Metabolic Equivalent of Task (MET) levels where light intensity is 1.6–2.9 METs, moderate is 3–5.9 METs and vigorous is ≥ 6 METs (Ainsworth et al., 2011). The duration of vigorous intensity activities was doubled to reflect the alternative ways of meeting the PA guidelines. A list of the intensity levels of the different sport and exercise activities is in the Supplementary material.

### 2.3 Sample

There were 4894 adult (age ≥ 16 years) responses to the 2013 SHeS. The decision to include 16–18 year olds was made to maintain comparability as they are considered adults in the UK health surveys and reported on as such, despite the adult UK PA guidelines applying to those aged 19 years upwards (Department of Health, 2011). We excluded cases if they reported implausible/incomplete values (over 10 h per day in one domain) (n = 9). If there were missing data for an individual sport or exercise activity, or for a whole domain, the contribution of this activity or domain was set to 0 rather than excluding the whole case.

The current analysis included the remaining 4885 adults. Activity status was determined by average reported weekly PA: those reporting no minutes of MVPA (n = 909), insufficiently active individuals reporting 1–149 min (n = 960) and active individuals reporting ≥ 150 min (n = 3016). Those reporting 0 min of MVPA were not included in any further analysis because the denominator of a percentage cannot be zero.

### 2.4 Statistical analyses

The relative proportions and the weekly minutes of MVPA of each domain were calculated for each individual who reported any MVPA (n = 3976). Linear regression analyses were used to assess differences in the absolute and relative contributions of the domains stratified by sex and activity status and split by age group (16–24, 25–34, 35–44, 54–64, 65 +). Differences in total MVPA were also assessed. We did not run regression analyses if the maximum relative contribution of the domain was < 10%.

Individuals who reported ≥ 150 min MVPA per week were analysed twice; (1) with occupational activity included; and (2) with occupational activity excluded (even if this took them under 150 min MVPA, although those who dropped to 0 min (n = 63) had to be excluded as the denominator could not be 0). This was because a low number of individuals reported a very high level of occupational PA, potentially distorting the findings; by conducting both analyses we could assess this effect. Only the relative contributions and total MVPA were reported and reanalysed using regression analyses as the exclusion of the 63 individuals barely altered the absolute contributions (a maximum of 6 min). No insufficiently active individuals reported any occupational activity.

All analyses were conducted in STATA/SE 14.0 using the “svyset” command to take into account the complex sampling design. This included using the weights provided by the SHeS to account for non-response bias and unequal selection probabilities to ensure reliable population estimates (Corbett et al., 2014).

## 3 Results

We found 64.3% of the sample (unweighted n = 3016) reported ≥ 150 min of weekly MVPA and therefore met the PA guidelines; 18.6% (unweighted n = 960) were insufficiently active reporting between 1 and 149.99 min of MVPA per week; 17.2% (unweighted n = 909) did not report any minutes of MVPA. As shown in Table 1, the proportion of adults meeting the PA guidelines decreased with age in both sexes. The proportion reporting 0 min of weekly MVPA increased with age in both sexes. We assessed the concurrent validity for our domain based approach by comparing to figures reported in the Scottish Health Survey 2013 main report and found our figures were within 0.1% (Hinchliffe, 2014). The minor discrepancies were due to our exclusion of implausible and incomplete cases.

**Fig. 1:**
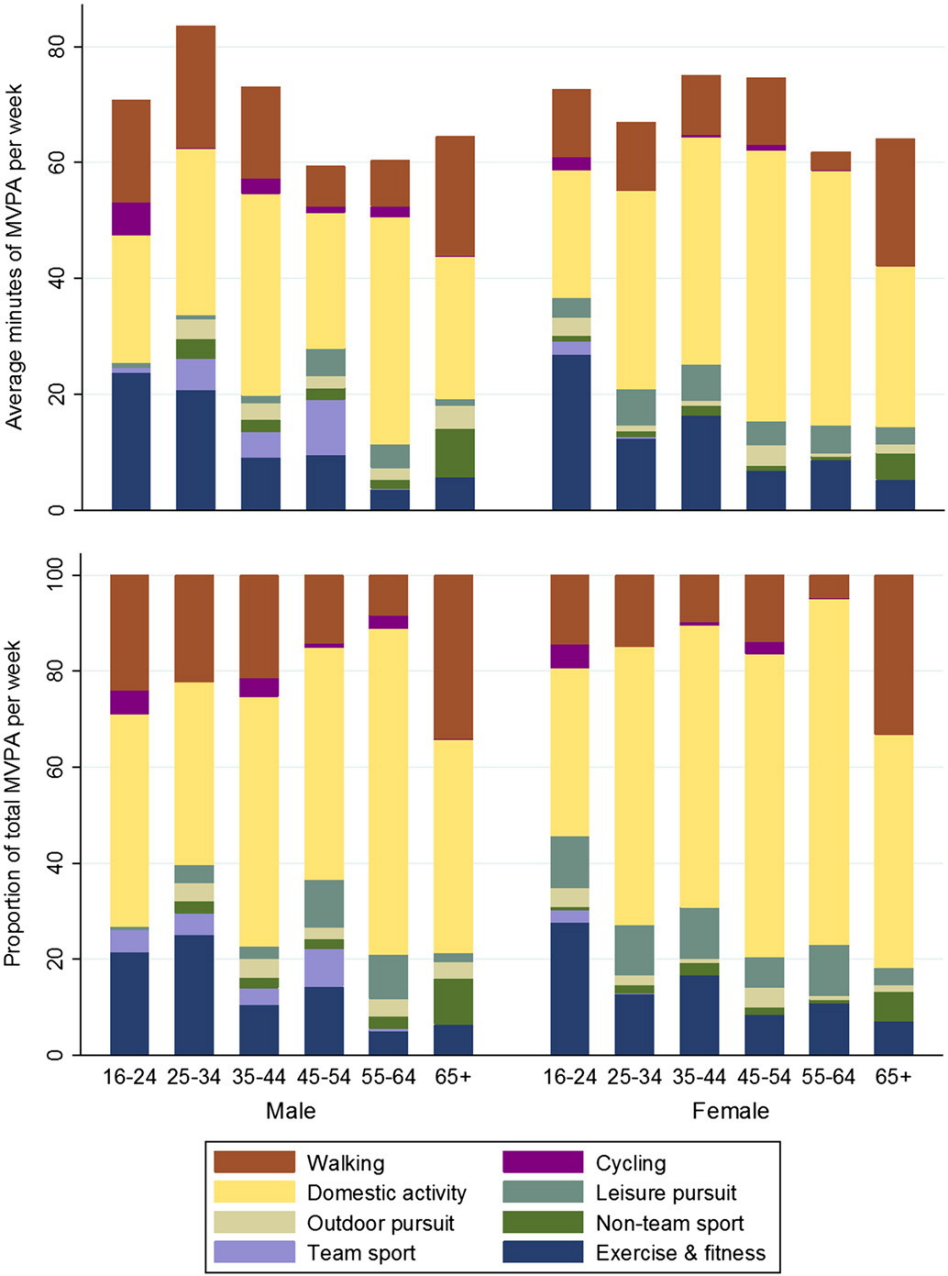
Domain-specific mean weekly minutes of moderate and vigorous physical activity (MVPA) and their respective relative contributions to total MVPA of adults in Scotland not did not meet the MVPA guidelines in 2013 (n = 960), by age category and sex.

Domestic activity was the most prevalent domain for both sexes in the insufficiently active category, accounting for between a third and three quarters of total MVPA across the age groups (Fig. 1). Exercise & fitness and walking accounted for most of the remainder, although the average weekly minutes were low (Table 2). There was a significant effect of age group on the absolute and relative contributions of the three main domains with the exception of the absolute contributions of domestic activity for men (all p < 0.05). In the case of domestic activity, this was due to fluctuations across the age groups rather than a clear trend. For exercise and fitness, the absolute and relative contributions to total MVPA gradually declined with age for both sexes, whereas for walking, the relative contributions were highest for both sexes in the 65 + category and the absolute contributions were only matched by younger men. Total MVPA did not vary by age group.

**Fig. 2:**
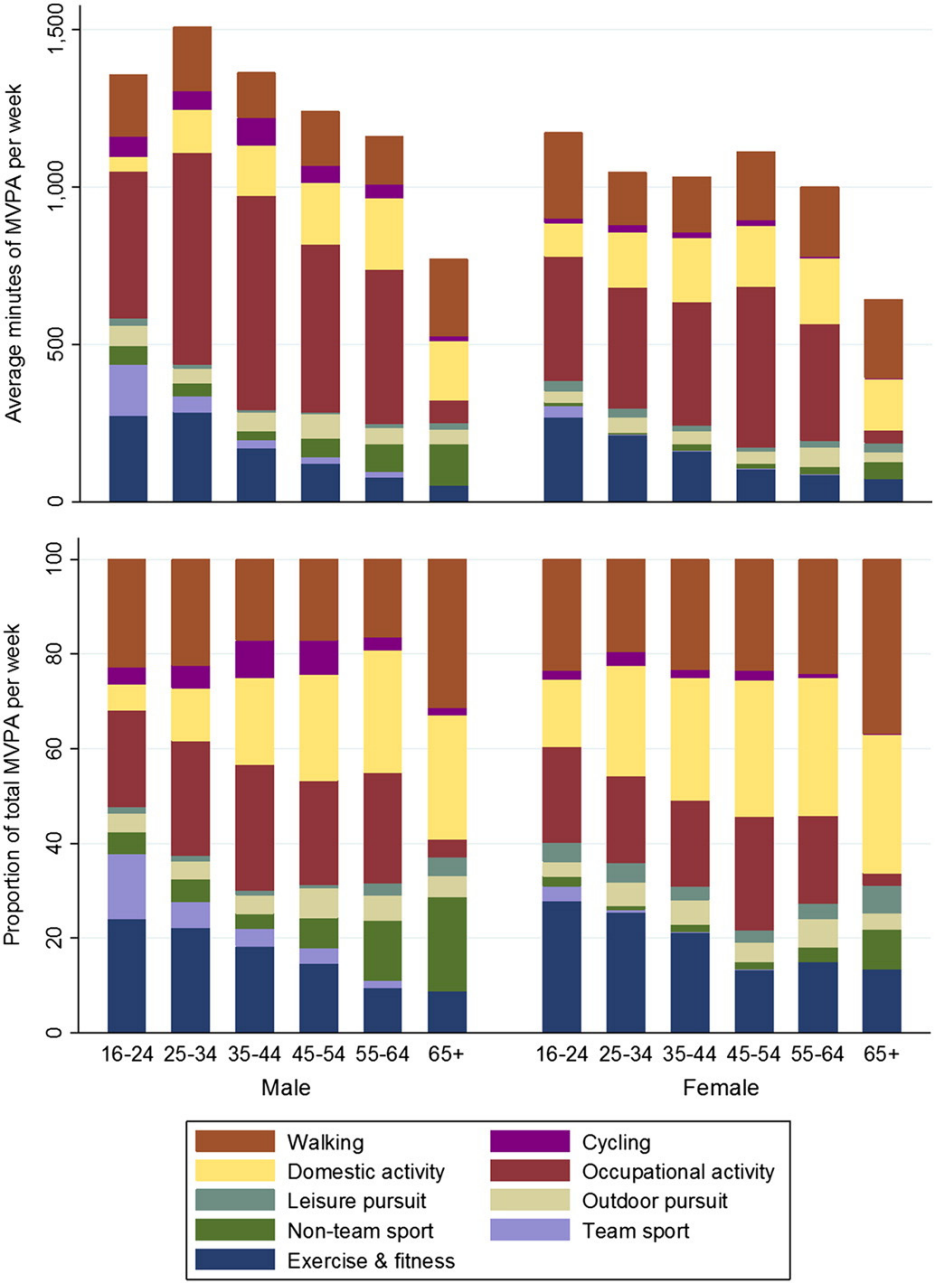
Domain-specific mean weekly minutes of moderate and vigorous physical activity (MVPA) and their respective relative contributions to total MVPA of adults in Scotland who met the MVPA guidelines in 2013 (n = 3016), by age category and sex.

Amongst adults who met the aerobic guidelines, occupational activity was the most prevalent domain for those under the age of 65 in both sexes accounting for 18–26% of all MVPA (Fig. 2, Table 3). Total weekly MVPA decreased with age for both men and women (p < 0.001).

**Fig. 3:**
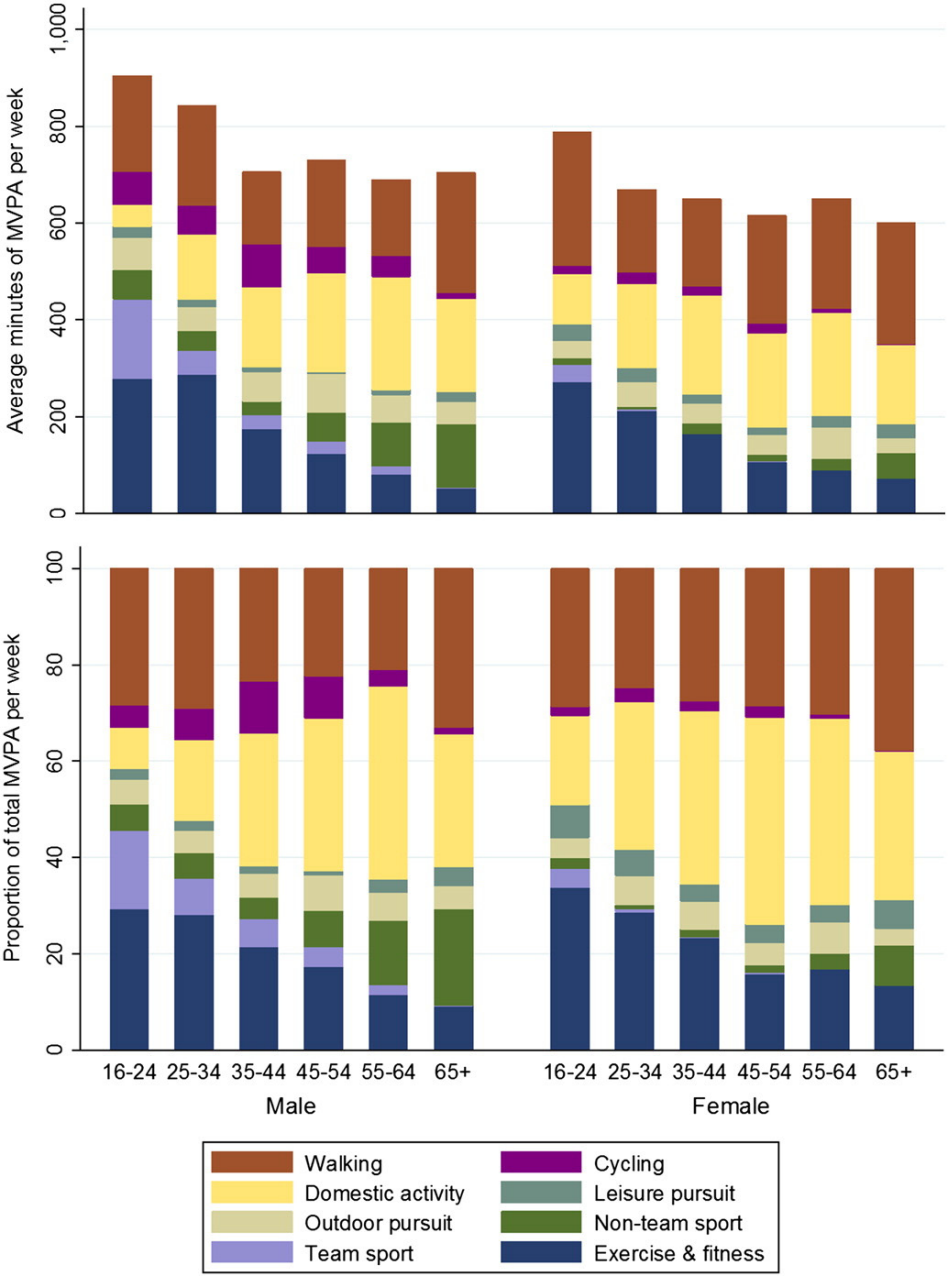
Domain-specific mean weekly minutes of moderate and vigorous physical activity (MVPA) and their respective relative contributions to total MVPA of adults in Scotland who met the MVPA guidelines in 2013 (n = 3016), excluding the domain of occupational activity, by age category and sex.

The high durations of occupational activity were due to around one quarter of those who met the guidelines (unweighted n = 741) reporting a large amount of activity in this domain (n = 414 reported over 2100 min/35 h per week). Therefore, total weekly MVPA and the relative contributions of the domains are presented excluding the domain of occupational activity (Fig. 3, Table 3). According to these data, there was no significant decline in total weekly MVPA by age (p > 0.05). Walking, domestic activity and exercise & fitness together accounted for around three quarters of all MVPA for both sexes. The absolute and relative contributions of these domains varied significantly by age group for both sexes, with the exception of the absolute contribution of walking in women. Exercise & fitness declined with age for both sexes whilst domestic activity increased. As with the insufficiently active, the over 65 s had highest relative contributions for walking and the absolute values were only exceeded by young women. Team and non-team sport accounted for between 5 and 20%, with the higher relative proportions amongst men in the youngest and oldest age groups.

## 4 Discussion

Our paper presents the first nationally representative domain-specific analysis of PA for adults in Scotland. We aimed to investigate the age-related variations in the domain-specific contributions to total MVPA by sex and activity status. We found significant variations in the most prevalent domains for men and women who met current aerobic guidelines and who were insufficiently active. We also found that, amongst those who met the guidelines, there was no evidence of a decline in total MVPA when occupational activity was excluded.

Occupational activity is challenging to assess and the method used in the SHeS inflates estimates and distorts analyses. All other domains are derived from the responses to specific questions on relevant activities. For occupational activity, individuals who report being ‘very physically active at work’ are allocated 40 or 20 h (for full or part time workers respectively) minus their reported sedentary time at work, of moderate activity per week, overwhelming all other domains. Those who report being ‘fairly active at work’ or other less active options are not allocated any occupational MVPA. Removing the domain of occupational activity highlights that for adults in Scotland who meet the guidelines, MVPA does not decline with increasing age as commonly thought (Nelson et al., 2007).

This is a novel finding as previous work has focussed on the reduction in the proportion meeting the guidelines with age, with or without the domain of occupational activity (Allender et al., 2008, Berger et al., 2005). These results are compatible: those who continue to meet the guidelines maintain MVPA levels. However, as age increases, a greater proportion report insufficient or no MVPA, a finding also reported in this study. This is a more nuanced view of how PA varies with age as it implies that a significant proportion are maintaining their MVPA levels — a positive message that should not be lost.

Whilst the cross sectional nature of these data prevents in-depth analysis of the retirement transition, this paper contributes to the literature surrounding changes in PA levels and domains at this stage of life (the average age of retirement in the UK was 63 years in 2010 (Office for National Statistics, 2012)). There is currently no consensus as to how retirement alters total PA although it is clear that it is modified by occupation type (Barnett et al., 2012). These data are also consistent with increases in exercise and leisure-time activity after retirement (Barnett et al., 2012), as the absolute and relative amounts of walking and non-team sport in those over 65 were amongst the highest reported in any age group. Further investigation showed this was mainly due to higher levels of golf and bowls in the older age groups.

This analysis also challenges the assumption that more intense activities are not relevant for the older ages. These data show that both active and insufficiently active older adults take part in activities in the domains of exercise & fitness and non-team sport, although walking and domestic activity are the major contributors to total MVPA. These findings are in line with recent data from national survey of Australian adults that showed walking participation increased with age, and that although participation in aerobics/fitness training decreased with age, it was still prevalent amongst adults aged 50 + (Eime et al., 2015). Our results support the current efforts in Scotland to develop and evaluate walking interventions (Macmillan et al., 2011, Mutrie et al., 2012) but are an important reminder not to place or encourage limits on the types of activity undertaken.

Analysis of PA by two strata of activity (the active and the insufficiently active) allows novel consideration by an important grouping variable. Analysis by domain elucidates how these levels are achieved. Domestic activity is the largest contributor to total MVPA amongst the insufficiently active. This group still takes part in walking, exercise & fitness, team and non-team sports but the average total durations are insufficient to meet PA recommendations. This suggests that policy for the insufficiently active could focus on increasing the duration of current activities, rather than the uptake of new activities.

Our findings have two main differences to those of Bélanger et al. (2011). Firstly, we found that non-team sport was a much greater contributor to total MVPA, particularly amongst older adults, compared to the results of Bélanger et al. (2011). This may demonstrate real differences in the countries' participation levels of the most prevalent sports in this category (golf, bowls and tennis). Or, it may be the result of some updates to the SHeS that occurred in 2012. These included the extension of the prompts for sport and exercise activities and the realignment of the intensities assigned in accordance with the latest MET compendium (Ainsworth et al., 2011). Overall, these changes led to higher reporting of sport and exercise activities and a net increase in the activities that count as MVPA. The new guideline also meant vigorous sports ‘counted double’ therefore increasing their contributions to total MVPA. Secondly, we reported lower relative contributions for walking across all ages than Bélanger et al. (2011) (approximately 10–20 percentage point differences). It is not clear whether this is a result of the small increases in other categories where the duration of vigorous activities has been doubled or whether this is a true difference.

The strengths of our study are that it is novel analysis for Scotland with policy implications. It is based on a nationally representative sample, reflecting the self-reported PA habits of adults in Scotland and provides comparable results to published analysis from England (Bélanger et al., 2011). The decision to exclude extreme implausible outliers was taken to maximise the comparisons with monitoring statistics and due to the nature of the data itself. The comprehensive nature of the questionnaire and the assumptions necessary to generate summary variables, such as doubling the duration of vigorous intensity activities to account for the flexibility in the guidelines (see Corbett et al. (2014) for details) appear to result in consistently higher total MVPA than might be considered typical. Given the convergent validity of the questionnaire has not been tested against an accepted gold standard, we cannot rule out the overestimation of MVPA levels and the proportion meeting the guidelines. However, there were no differences in the conclusions when the analyses were re-run with a stricter approach to outliers (excluded all with average daily MVPA > 10 h). Similarly, there were no differences to the conclusions when the analysis was re-run excluding cases with missing data on any items from the entire analysis.

We did not divide the ‘65 +’ age group further in order to maintain sample size and maintain comparability to Bélanger et al. (2011). Additionally, the current PA guidelines provide recommendations for those over 65 as one group and therefore this division has policy relevance. However, we acknowledge that this age group is likely to be heterogeneous in their PA behaviours and a further division at age 75 would give further insight into the population changes in the domains of activity a decade after the typical retirement age. In any case, analysis of this group individually should be approached with caution as the SHeS is only representative of the population in private houses and does not include individuals who reside in residential care homes. Therefore the sample in this age group is potentially atypical of the population as it is likely to exclude frailer individuals.

The interpretation of the results is limited by the cross sectional nature of the study. Furthermore, the sample sizes for some age groups amongst the inactive are very low, potentially limiting their ability to reflect the wider population. This analysis would be improved if active travel could have been considered as a separate domain. Despite active travel being a government priority (Transport Scotland, 2014), there is no way of determining whether the walking or cycling reported in the SHeS falls under this category and how this varies by age group.

These findings have implications for informing PA policy and promotion in Scotland. They show the need to increase PA across all domains. Walking and occupational activity were the most prevalent domains and should receive at least equal attention as sport and exercise. An important domain will be walking due to its accessibility across age and social inequality as promoted by the National Walking Strategy (The Scottish Government, 2014a). Analysis has shown walking, even at low levels, can significantly reduce risk for all-cause mortality (Kelly et al., 2014) and increase health-related fitness (Kelly et al., 2011). However, it is evident that sports and exercise are still acceptable across age and gender, even in the insufficiently active. We feel this suggests that nuanced policy could focus onto increasing engagement in existing activities, rather than policy to promote new activities.

## 5 Conclusions

In conclusion, this paper provides nationally representative data for Scotland on how the domains of PA vary by age for both sexes and different activity statuses. The results highlight how the measurement of occupational activity distorts our understanding of the situation, as once the domain is excluded from analyses, total MVPA did not decline across the age groups for those who continue to meet the PA guidelines. The findings have implications for policy and practice: these data provide support for the emphasis being placed on the National Walking Strategy (CTC Scotland, 2015) and indicate that policymakers should be more sensitive to the range of domains in which PA takes place and the variations of participation across the life-course and between sexes. It is likely that the major differences between Scottish and English data are due to methodological variations but this may warrant further confirmation. The current findings should be interpreted in light of the fact that there has been no assessment of the convergent validity of the SHeS PA questionnaire against accepted gold standards; future research should address this. We are confident our analyses offer a real starting point for policy makers to examine if their interventions are promoting the right activities to the right people at the right stage of life.

## Conflict of interest/funding source

The authors declare that there are no conflicts of interest. Tessa Strain is funded by a College Research PhD studentship from the University of Edinburgh. The posts of Charlie Foster and Nick Townsend are funded by the British Heart Foundation (grant number for Nick Townsend 006/P&C/CORE/2013/OXFSTATS).

## Transparency document

Transparency document.

## Figures and Tables

**Table 1:** Percentage of adults in Scotland who report no moderate and vigorous physical activity (MVPA), insufficient MVPA or sufficient MVPA to meet the MVPA guidelines^a^ in 2013, by age category and sex.

Average weekly minutes of MVPA	Men							Women						
16–24	25–34	35–45	44–54	54–65	65 +	All	16–24	25–34	35–45	44–54	54–65	65 +	All
0	5.3	4.7	11.1	10.1	20.6	34.1	14.9	10.0	7.5	10.7	15.0	23.8	39.2	19.2
1–149.99 (insufficiently active)	7.2	11.6	15.2	13.6	18.5	19.0	14.4	19.7	19.2	20.4	18.8	27.0	27.3	22.4
150 + (active)	87.5	83.7	73.7	76.3	60.9	46.9	70.6	70.3	73.4	68.8	66.2	49.1	33.5	58.4
Unweighted bases	204	311	339	394	353	534	2135	241	419	431	538	442	679	2750
Weighted bases^b^	334	370	387	437	366	446	2340	332	389	411	459	383	567	2542

a 150 min moderate activity, or 75 min of vigorous activity or equivalent combination per week.

b Sample weights are applied to account for non-response bias and unequal selection probabilities.

**Table 2:** Age-related variations in the domain-specific minutes of weekly moderate and vigorous physical activity (MVPA) and their respective relative contributions to total MVPA for adults in Scotland who did not meet the MVPA guidelines^a^ in 2013, by sex.

	Men															
16–24		25–34		35–44		45–54		55–64		65 +		All		Main effect of age	
Mean min MVPA	Relative contribution	Mean min MVPA	Relative contribution	Mean min MVPA	Relative contribution	Mean min MVPA	Relative contribution	Mean min MVPA	Relative contribution	Mean min MVPA	Relative contribution	Mean min MVPA	Relative contribution	Mean min MVPA	Relative contribution
Exercise and fitness	23.6	21.5	20.7	25.1	9.1	10.4	9.6	14.2	3.5	5.1	5.7	6.4	9.7	11.7	⁎	⁎
Team sport	1.0	4.6	5.5	4.5	4.3	3.5	9.6	7.8	0.2	0.5	0.0	0.0	3.2	3.0	–	–
Non-team sport	0.0	0.0	3.3	2.4	2.2	2.1	2.0	2.2	1.5	2.4	8.4	9.5	3.6	3.9	–	–
Outdoor pursuit	0.0	0.0	3.5	3.8	2.8	3.9	2.1	2.3	2.1	3.8	4.1	3.4	2.7	3.2	–	–
Leisure pursuit	0.8	0.8	0.6	3.8	1.4	2.6	4.7	9.9	4.0	9.2	1.1	2.0	2.3	5.1	–	–
Occupational	0.0	0.0	0.0	0.0	0.0	0.0	0.0	0.0	0.0	0.0	0.0	0.0	0.0	0.0	–	–
Domestic	21.9	44.2	28.7	38.0	34.8	52.1	23.4	48.4	39.2	67.8	24.5	44.4	29.4	50.3	ns	⁎
Cycling	5.8	4.9	0.1	0.1	2.6	3.9	1.0	1.0	1.9	2.9	0.2	0.1	1.5	1.8	–	–
Walking	17.6	24.1	21.1	22.2	15.9	21.5	7.0	14.1	8.0	8.3	20.6	34.2	14.7	21.0	⁎	⁎⁎
Total	70.8		83.6		73.1		59.3		60.4		64.5		67.1		ns	
Unweighted bases	18		40		48		58		56		108		328			
Weighted bases^b^	24		43		59		59		68		85		338			

	Women															
Exercise and fitness	26.8	27.8	12.3	12.8	16.4	16.6	6.8	8.3	8.7	10.9	5.2	7.1	11.2	12.5	⁎⁎	⁎
Team sport	2.3	2.3	0.3	0.2	0.0	0.0	0.0	0.0	0.0	0.0	0.0	0.0	0.3	0.3	–	–
Non-team sport	0.9	0.8	0.9	1.5	1.7	2.7	0.9	1.5	0.6	0.7	4.7	6.1	2.0	2.7	–	–
Outdoor pursuit	3.2	3.8	1.0	2.0	0.8	0.7	3.5	4.2	0.5	0.7	1.4	1.3	1.6	1.9	–	–
Leisure pursuit	3.3	10.8	6.3	10.6	6.2	10.8	4.0	6.3	4.8	10.7	3.0	3.7	4.4	8.1	ns	⁎
Occupational	0.0	0.0	0.0	0.0	0.0	0.0	0.0	0.0	0.0	0.0	0.0	0.0	0.0	0.0	–	–
Domestic	22.0	34.9	34.2	57.9	39.4	58.9	46.8	63.3	43.8	72.0	27.6	48.5	35.4	56.2	⁎⁎	⁎⁎
Cycling	2.4	5.1	0.0	0.1	0.4	0.5	1.0	2.5	0.1	0.3	0.0	0.0	0.5	1.1	–	–
Walking	11.5	14.3	11.8	14.9	10.2	9.9	11.5	13.9	3.2	4.7	22.1	33.3	12.7	17.1	⁎⁎	⁎⁎
Total	72.5		66.9		75.0		74.6		61.7		64.2		68.2		ns	
Unweighted bases	46		83		92		109		110		192		632			
Weighted bases^b^	65		75		84		86		104		155		569			

–: Regression not performed as relative contribution does not exceeded 10%.

ns: Not significant at p < 0.05.

a 150 min moderate activity, or 75 min of vigorous activity or equivalent combination per week.

b Sample weights are applied to account for non-response bias and unequal selection probabilities.

⁎ Significant at p < 0.05.

⁎⁎ Significant at p < 0.01.

**Table 3:** Age-related variations in the domain-specific minutes of weekly moderate and vigorous physical activity (MVPA) and their respective relative contributions to total MVPA, with and without the domain of occupational activity, for adults in Scotland who met the MVPA guidelines^a^ in 2013, by sex.

	Men																							
16–24			25–34			35–44			45–54			55–64			65 +			All			Main effect of age		
Mean min MVPA	Relative contribution	Relative contribution excl. occupational	Mean min MVPA	Relative contribution	Relative contribution excl. occupational	Mean min MVPA	Relative contribution	Relative contribution excl. occupational	Mean min MVPA	Relative contribution	Relative contribution excl. occupational	Mean min MVPA	Relative contribution	Relative contribution excl. occupational	Mean min MVPA	Relative contribution	Relative contribution excl. occupational	Mean min MVPA	Relative contribution	Relative contribution excl. occupational	Mean min MVPA	Relative contribution	Relative contribution excl. occupational
Exercise and fitness	273.4	24.0	29.3	284.7	22.0	28.0	169.9	18.1	21.6	119.0	14.6	17.4	77.9	9.5	11.5	52.2	8.8	9.2	172.1	16.8	20.4	⁎⁎	⁎⁎	⁎⁎
Team sport	160.3	13.8	16.2	49.1	5.7	7.6	25.9	3.8	5.8	24.1	3.2	4.1	17.0	1.5	2.1	0.8	0.1	0.1	49.3	5.0	6.4	⁎⁎	⁎⁎	⁎⁎
Non-team sport	59.7	4.5	5.5	41.5	4.7	5.3	28.7	3.2	4.4	57.5	6.5	7.6	87.1	12.8	13.3	129.3	19.7	20.1	63.0	7.7	8.6	⁎⁎	⁎⁎	⁎⁎
Outdoor pursuit	65.2	4.0	5.2	47.6	3.7	4.6	59.8	3.9	4.8	76.9	6.3	7.1	54.2	5.4	5.7	46.9	4.7	4.7	59.5	4.7	5.4	–	–	–
Leisure pursuit	22.8	1.4	2.2	14.4	1.3	2.1	8.1	0.9	1.6	5.5	0.7	1.0	10.5	2.5	3.0	20.4	3.8	3.9	13.2	1.6	2.2	–	–	–
Occupational	468.1	20.4	N/A	671.6	24.3	N/A	679.4	26.6	N/A	534.5	22.1	N/A	491.6	23.3	N/A	73.4	3.8	N/A	509.3	20.8	N/A	⁎⁎	⁎⁎	N/A
Domestic	44.3	5.5	8.6	134.9	11.0	16.7	160.9	18.3	27.7	196.0	22.3	31.8	224.9	25.8	40.0	188.5	26.2	27.6	154.6	17.5	24.7	⁎⁎	⁎⁎	⁎⁎
Cycling	65.5	3.5	4.7	58.5	4.9	6.6	86.2	8.0	10.7	53.0	7.3	8.7	43.7	2.8	3.4	12.5	1.4	1.4	55.6	4.9	6.3	⁎⁎	⁎⁎	⁎⁎
Walking	196.1	22.9	28.4	205.7	22.4	29.1	144.8	17.1	23.4	173.2	17.0	22.3	152.2	16.5	21.0	246.6	31.5	33.0	184.9	20.8	26.0	⁎	⁎⁎	⁎⁎
Total including occupational	1355.4			1507.9			1363.6			1239.6			1159.2			770.6			1261.6			⁎⁎		
Total excluding occupational^b^	904.5			842.9			706.0			730.7			690.1			704.3			769.9			ns		
Unweighted bases	176			258			255			292			219			245			1445					
Weighted bases^c^	293			310			285			333			223			209			1653					

	Women																							
Exercise and fitness	267.5	27.8	33.8	210.5	25.4	28.6	160.0	21.2	23.4	104.4	13.2	15.8	86.6	15.0	16.8	72.8	13.5	13.5	154.7	19.6	22.4	⁎⁎	⁎⁎	⁎⁎
Team sport	34.5	3.2	3.9	3.6	0.5	0.7	1.6	0.1	0.1	1.9	0.2	0.3	0.6	0.1	0.1	0.1	0.0	0.0	6.9	0.7	0.8	–	–	–
Non-team sport	13.7	2.0	2.3	4.7	0.8	0.8	21.0	1.5	1.6	12.8	1.4	1.6	23.1	3.0	3.1	52.9	8.3	8.3	19.4	2.5	2.6	–	–	–
Outdoor pursuit	36.2	3.0	4.0	50.4	5.0	5.9	40.4	5.2	5.7	39.2	4.2	4.6	62.5	6.0	6.6	29.7	3.5	3.5	42.8	4.5	5.1	–	–	–
Leisure pursuit	32.2	4.0	7.0	28.0	4.1	5.5	17.7	2.9	3.5	15.0	2.6	3.8	21.9	3.3	3.6	29.3	5.9	5.9	23.4	3.7	4.8	–	–	–
Occupational	396.0	20.3	N/A	382.8	18.3	N/A	393.3	18.1	N/A	510.6	24.1	N/A	370.8	18.5	N/A	41.4	2.6	N/A	367.8	17.8	N/A	⁎⁎	⁎⁎	N/A
Domestic	103.8	14.2	18.5	173.9	23.3	30.6	203.1	25.8	36.0	190.7	28.6	43.1	207.1	29.1	38.7	164.2	29.3	30.8	174.9	24.9	33.3	⁎⁎	⁎⁎	⁎⁎
Cycling	16.6	1.9	2.0	23.4	2.9	3.0	17.8	1.8	2.1	19.6	2.1	2.3	7.1	0.8	1.0	1.1	0.1	0.1	15.6	1.7	1.9	–	–	–
Walking	271.9	23.6	28.6	169.3	19.6	24.8	177.0	23.4	27.6	218.0	23.5	28.6	220.0	24.2	30.2	251.8	36.8	37.9	213.8	24.6	29.1	ns	⁎⁎	⁎⁎
Total including occupational	1172.3			1046.5			1031.9			1112.2			999.7			643.2			1019.3			⁎⁎		
Total excluding occupational^b^	788.5			669.2			649.5			616.5			649.5			601.8			662.3			ns		
Unweighted bases	166			307			294			350			228			226			1571					
Weighted bases^c^	223			286			283			304			188			190			1484					

–:Regression not performed as relative contribution does not exceeded 10%.

ns: Not significant at p < 0.05.

a 150 min moderate activity, or 75 min of vigorous activity or equivalent combination per week.

b Totals may not add up due to the exclusion of 63 individuals who dropped to 0 min of MVPA per week once occupational was excluded. The bases shown refer to the sample sizes for the calculations including occupational activity.

c Sample weights are applied to account for non-response bias and unequal selection probabilities.

⁎ Significant at p < 0.05.

⁎⁎ Significant at p < 0.01.

## Appendix A Supplementary data

See Supplementary material for information on the allocation of sport and exercise activities to sub-domains and their intensity levels.
