# Supplementary material for: Age-related comparisons by sex in the domains of aerobic physical activity for adults in Scotland
Source: Prev Med Rep. 2015 Dec 30;3:90–7. doi: 10.1016/j.pmedr.2015.12.013 (PMC4733093; doi:10.1016/j.pmedr.2015.12.013)
Supplement: Supplementary file 2 — See Supplementary material for information on the allocation of sport and exercise activities to sub-domains and their intensity levels. [file mmc2.pdf]

**Supplementary Materials**

**Exercise & fitness**
Workout at gym/Exercise bike/Weight training*, Swimming^, Running/jogging*, Walking/jogging on treadmill˅, Exercise (e.g. press ups, sit ups)*, Aerobics/Keep fit/Gymnastics/Dance for Fitness*, Yoga/Pilates˅, Aquarobics*, Circuit training*, Skipping^

**Team sports**
Football/Rugby^, Basketball*, Netball*, Cricket, Rounders, Curling, Hockey^, Rowing*, Volleyball*, Shinty^

**Non-team sports**
Badminton/Tennis*, Squash^, Martial arts including Tai Chi*, Golf, Bowls, Archery, Croquet, Swing ball, Table tennis, Boxing*, Kick boxing^, Athletics*

**Outdoor pursuit**
Water skiing, Skiing/Snowbarding*, Horse Riding*, Canoeing/Kayaking*, Climbing*, Hillwalking/Rambling*, Hang-gliding/Parachuting, Orienteering^, Skateboarding/Inline skating*, Rollerblading*, Sailing/Windsurfing*, Surfboarding/Bodyboarding

**Leisure pursuit**
Swimming, Dancing*, Drumming, Fishing/Angling˅, Ice skating*, Motor Sports, Powerboating/Jetskiing*, Subaqua^

**Cycling**
Cycling*

The additional question to determine intensity level was:

During the past four weeks, was the effort of [*activity name*] usually enough to make you breathe faster, feel warmer, or sweat? Yes/No

The intensities of the activities were calculated accordingly:

^There activities were always counted as vigorous intensity.

*These activities were counted as vigorous intensity the response to the additional question was ‘yes’. They were counted as moderate if the response was ‘no’.

˅These activities were counted as moderate intensity if the response to the additional question was ‘yes’. They were counted as light intensity (i.e. not included in analysis) if the response was ‘no’.

All other activities were always counted as moderate intensity.
